# Natural and herbal compounds targeting breast cancer, a review based on cancer stem cells

**DOI:** 10.22038/ijbms.2020.43745.10270

**Published:** 2020-08

**Authors:** Azam Bozorgi, Saber Khazaei, Abbasali Khademi, Mozafar Khazaei

**Affiliations:** 1 Fertility and Infertility Research Center, Health Technology Institute, Kermanshah University of Medical Sciences, Kermanshah, Iran; 2 Dental Research Center, Dental Research Institute, Isfahan University of Medical Sciences, Isfahan, Iran

**Keywords:** Breast neoplasm, Neoplasm, Phenolic compounds, Stem cells, Therapy resistance

## Abstract

Cancer stem cells (CSCs) are known as the major reason for therapy resistance. Recently, natural herbal compounds are suggested to have a significant role in inhibiting the breast cancer stem cells (BCSCs). The aim of this study was to explore the effective natural herbal compounds against BCSCs.This review article was designed based on the BCSCs, mechanisms of therapy resistance and natural herbal compounds effective to inhibit their activity. Therefore, Science direct, PubMed and Scopus databases were explored and related original articles were investigated from 2010 to 2019. BCSCs use different mechanisms including special membrane transporters, anti-apoptotic, pro-survival, and self-renewal- related signaling pathways. Natural herbal compounds could disturb these mechanisms, therefore may inhibit or eradicate the BCSCs. Studies show that a broad range of plants, either as a food or medicine, contain anti-cancer agents that phenolic components and their different derivatives share a large quantity. Natural herbal compounds play a pivotal role in the eradication of BCSCs, through the inhibition of biological activities and induction of apoptosis. Although it is necessary to conduct more clinical investigation.

## Introduction

Breast cancer (BC) is one of the most challenging forms of cancer because of its high prevalence rate (about 1.7 million new cases diagnosed) and also a high rate of death reports among women (approximately 0.5 million death cases) (1). International Agency for Research on Cancer has reported that approximately 2.1 million cases diagnosed in 2018 (2). Although in the recent decade, different therapeutic techniques including surgery, radio/chemo/ hormonal therapy or targeted therapies have been presented (3), and in spite of the development of innovative anti-cancer drugs acting effectively, patients suffer from consequences of relapse and metastasis (4). 

Through all of the possible reasons that have been suggested to explain the occurrence of metastasis, the theory of the presence of breast cancer stem cells (BCSCs) has been faced with more success. BCSCs are defined with unique properties including high proliferation, the ability to self-renewal and the generation of heterogeneous lineages (5). This great achievement, along with studies that have been made in the field of natural compounds found in diet plants and herbal medicine, has tended scientists to explore the role of these compounds in cancer therapy with special attention to target CSCs. 

Nowadays, it is well known that natural compounds, generally obtained from plants are useful to treat some of the human diseases (6) including cancer. However, scientists have explored the role of marine natural compounds as anti-cancer agents (7), but most of the studies have been established based on herbal compounds (8). Recently, in addition to different types of normal stem cells and their characteristics and applications in medicine that have been investigated (9), different aspects of CSCs especially BCSCs characteristics have been reviewed by scientists (10). In this article, in addition to the elucidation of the role of BCSCs to therapy resistance, natural herbal compounds against BCSCs are also reviewed.

***Therapy resistance, facing issue to breast cancer treatment***

**Figure 1:**
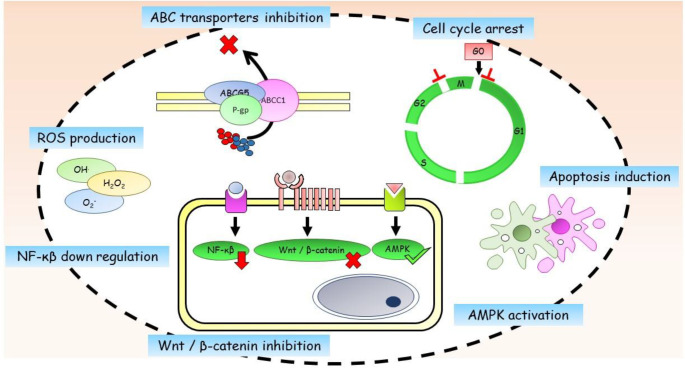
Mechanisms of therapy resistance in breast cancer stem cells

Although most chemotherapeutic drugs decline the size of tumors significantly (11), they fail to vanish it completely. As a result, the tumor resists to treatment and relapses. Proving the existence of CSCs in different cancers such as breast cancer (12), it has been demonstrated that chemo/radiotherapy interventions are unable to kill CSCs efficiently (13). Several studies state the inefficiency of chemotherapy (14) and radiotherapy (15) to eradicate CSCs and increased CSCs content of tumors following chemotherapy (16). Possible reasons for therapy resistance are listed below and are shown in Figure 1.

***Active anti-apoptotic pathways***

The apoptotic response of CSCs to treatment strongly depends on the equilibrium between pro-apoptotic and anti-apoptotic molecules that are expressed by CSCs. Pro-apoptotic molecules that are expressed including Bcl-2 homologous antagonist killer (BAK), Bcl-2 associated X protein (BAX) and Bcl-2 associated death promoter (BAD). In contrast, anti-apoptotic molecules belonging to the Bcl-2 family (BCL2, MCL1, BCL- XL) regulate the permeability of the outer membrane of the mitochondrion (17). Overexpression of anti-apoptotic molecules within the CSCs leads to therapy resistance. Suppressing the activity of these molecules targets CSCs effectively (18, 19).

***Cancer stem cell niche***

Within the tumor, CSCs reside in special environmental conditions, where they are not only affected by local physico/chemical factors (oxygen, PH, nutrients) but also they interact with other immune cells or fibroblasts existing on the same site. This special environmental condition is known as “niche”. External stimuli could influence niche severely. For example, radio/chemotherapy leads to the activation of interleukin6 (IL6) by niche elements. IL6, in turn, activates the nuclear factor kappa-light-chain-enhancer of activated B cells (STAT3/NF-Kβ) signaling pathway, which is related to increased BCSC population as well as resistance to Trastuzumab (20). Hypoxic niche has a key role in therapy resistance because of less production of reactive oxygen species (ROS) (21), induction of quiescence state of CSC (22), and eventually releasing the hypoxia-inducible factors 1 and 2 (HIF1, HIF2) (23).

***ATP- dependent drug efflux***

CSCs possess a thrifty and useful mechanism to elude from applied stress due to the cytotoxic drugs administered to the cells during chemotherapy. Chemotherapeutic drugs have high toxicity on CSCs, but the best answer to how CSCs resist against these agents is that CSCs have an advanced transport system in which ATP is consumed and cytotoxic drugs are pumped outside of the cell actively. The fundamental components of this membrane port are ATP- binding cassette (ABC) proteins, a family of membrane proteins with three well-known members involved in CSCs drug resistance, p- glycoprotein (P- gp), breast cancer resistant protein (BCRP), and multidrug-resistant protein 1 (MDR1). Therefore, ABC proteins lead to resistance to different types of chemotherapeutic agents such as antimetabolites, topoisomerase, and tyrosine kinase inhibitors and taxanes (24). 

***Pro- survival signaling pathways***

Different signaling pathways are contributed to maintaining self-renewal and differentiation of CSCs. These pathways seem to be somewhat responsible for resistance to therapy, therefore could be regarded as new therapeutic targets. The most important pathways are Notch and Hedgehog (25), as well as the Wnt pathway that play an important role to maintain the undifferentiated state and self-renewal conditions of BCSCs (26).

***DNA repair and ROS scavenging systems***

Radio/chemotherapy treatments lead to DNA damage and following tumor cell death. Instead, CSCs’ response to DNA damage appears as activation of ataxia-telangiectasia-mutated-and-Rad3-related kinase- checkpoint kinase 1 and 2 (ATR-ChK1 and ATM-ChK2) kinase signaling pathways. At the next step, cell cycle progress is suppressed and conditions are provided to DNA repair (27). Also, radiotherapy leads to the production of potentially lethal oxygen radicals known as ROS within the cell. BCSC’s strategy to evade cell death is an efficient ROS removal system in which following irradiation, little quantities of ROS are produced. Also, enzymes involving in ROS scavengings such as catalase, superoxide dismutase (SOD), and glutathione peroxidase are up-regulated in BCSCs (28). 

***Natural herbal compounds, future anti-cancer drugs with a different prospect***

**Figure 2:**
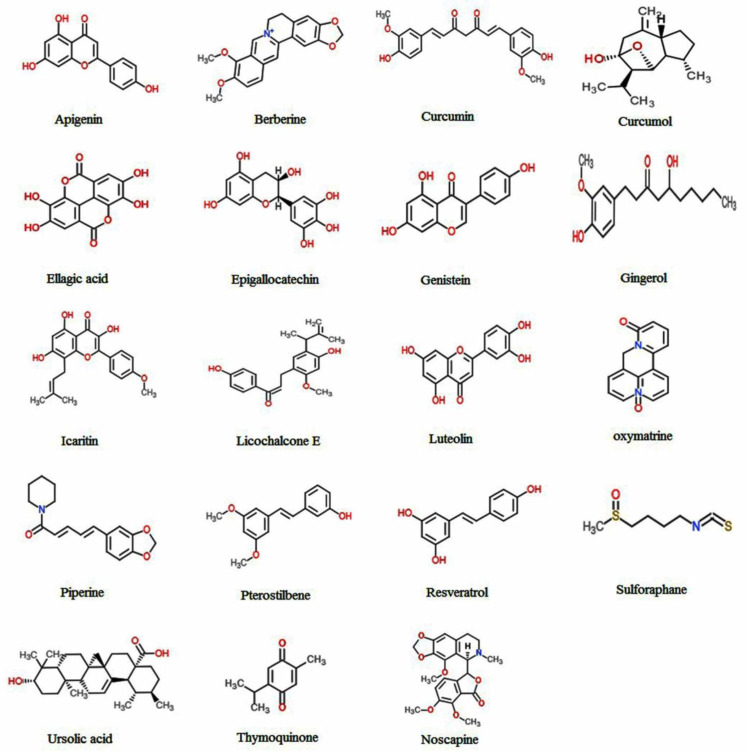
Chemical structures of natural herbal compounds with anti-cancer and anti-cancer stem cell effects

Recently, extensive studies about breast cancer, verifying the presence of BCSCs (10) and therapy resistance issues altogether, have forced scientists to recognize more effective anti-cancer drugs. Isolation, identification and inspecting the therapeutic effects of natural herbal compounds against cancer is a fascinating field of research. The advantage of these compounds is that they are tolerable and also could be added to the dietary regimen. As well, phytochemical compounds are used to inhibit tumor growth or to diminish the possibility of tumor relapse (29, 30). Some of the herbal compounds that have been investigated so far to suppress the BCSCs are listed below. The chemical structure and mechanism of action of these compounds are shown in Figure 2 and Table 1, respectively. 

***Phenolic compounds and their derivatives***

Phenolic compounds are natural herbal compounds derived from phenylalanine and tyrosine, which are found in food sources extensively [reviewed by Weng and Yen (31)]. Astragalin as a phenolic compound exists in some medicinal plants (32). Their chemical structures differ, but typically pose an aromatic ring with one or two hydroxyl groups. According to the number of aromatic rings and functional groups, phenolic compounds are divided into phenolic acids, monophenols, and polyphenols. Phenolic derivatives are found in a broad range that among of them simple phenols, ﬂavonoids, stilbene, phenylpropanoids, and benzoic acid derivatives are found in various types of plants and nutrients.

***Apigenin***

Apigenin is a natural flavonoid found in a great number of vegetables and fruits such as French peas, garlic, cabbage, bell pepper and many others (33, 34). Also, Apigenin has been found as an effective element of medical herbs such as *Salvia officinalis* (35), *Turnera aphrodisiaca* (36), *Lawsonia inermis* (37), *Ocimum basilicum* and *Tamarindus indica *(38). Apigenin has acquired more attention because of its preventive effects on cancer (39) and diverse effects including antioxidant, ROS removal, antiviral, anti-inflammatory and anti-mutagenic roles that have been evaluated in different systems of mammalian (40). 

The role of Apigenin in the inhibition of ABC transporters (p- glycoprotein and ABCB5 transporters) shows that it attaches to the ATP- binding domain of transporters, preventing the ATP attachment; therefore, the required energy to efflux the cytotoxic drugs is not provided. In addition, the activity of Apigenin does not interfere with classical strategies of multidrug resistance, so it seems that it could be conquered to multidrug resistance of different tumors (41). Also, it was shown that Apigenin is able to block tumor necrosis factor-alpha (TNFα) pathway in MDA- MB231 cancer cells; therefore, the following release of chemokines such as CCL2, granulocyte-macrophage colony-stimulating factor (GMCSF), IL-1α and IL-6 is inhibited. These pro-inflammatory chemokines have an important role in tumor growth and metastatic invasion (42).

 A study showed that Apigenin suppressed the migration of prostate CSCs via down-regulating matrix metalloproteinase 2, 9 (MMP2,9), slug and snail. Also, Apigenin induces apoptosis through an extrinsic pathway, which is associated with the expression of caspases 3 and 8 (43). Synergic anti-cancer effects of Apigenin and chrysin, a flavonoid with structural similarity to Apigenin, were evaluated. It was shown that both Apigenin and chrysin decreased MDA-MB-231 cell viability and motility, and induced apoptosis via down-regulating the expression of low-density lipoprotein receptor related-protein 6 (LRP6) and S- phase kinase-associated protein 2 (Skp2) (44). 

Apigenin could reverse drug resistance of adriamycin-resistant MCF7 cancer cells, which is associated with the arrest of cells in the sub-G0/G1 phase and increased apoptosis. It seems that the suppression of drug resistance by Apigenin depends on the blockade of the signal transducer and activator of transcription 3 (STAT3) signaling pathway (45). A similar data was previously reported in which Apigenin decreased the expression of phospho Janus Kinase 1/2 (phospho-JAK1/ JAK2), phospho-STAT3, and STAT3-dependent luciferase reporter gene activity in human epidermal growth factor receptor 2 (HER2)- expressing BT- 474 cancer cells (46). Li *et al.* demonstrated that Apigenin significantly suppressed the proliferation and migration of triple-negative breast cancer (TNBC) cells and inhibited stemness features of TNBC cells in both *in vitro* and *in vivo* assays. It seemed that Apigenin decreased yes-associated protein/ transcriptional coactivator with PDZ-binding motif (YAP/TAZ) activity and the expression of target genes, such as connective tissue growth factor (CTGF) and Cystein rich 61 (CYR61). They also showed that Apigenin disrupted the YAP/TAZ-TEADs (TEA domain transcription factors) protein-protein interaction and decreased expression of TAZ sensitized TNBC cells to Apigenin treatment (47).

***Berberine***

Berberine is an alkaloid derivative and a member of the *Berberidaceae* family, which is found in medicinal herbs *Coptis chinensis* and *Hydrastis canadensis* with anti-bacterial (48), anti-inflammatory (49) and verified anti-cancer properties (50, 51). It has been reported that berberine-containing liposomes pass through the membrane of CSCs, inhibit ABC transporters and finally assemble within the mitochondria. Also, berberine leads to activation of pro-apoptotic protein BAX, inhibition of anti-apoptotic protein Bcl-2, increased the permeability of the mitochondrial membrane, the release of cytochrome C and the activation of caspases 3, and 9 (52, 53). 

Berberine suppresses MCF7 and ZR- 75- 30 cell growth and migration through reducing the expression of ephrin-B2 and inhibition of MMP2 and 9, respectively (54). One study suggests that migration- inhibitory effect of berberine in MCF7 cancer cells is due to the down-regulation of chemokine receptors 4 (CXCR4), 6 (CCR6), and 9 (CCR9) (55). A combination of berberine with cisplatin suppressed MCF7 cell growth and induced apoptosis through up-regulation of caspases 3, and 9 and down-regulation of Bcl-2 (56).

***Curcumin and curcumol***

Curcumin is a polyphenolic element of turmeric, an Indian spice used to produce mustard and curry (57). Curcumin bears antioxidant and anti-inflammatory properties (57, 58). It has been shown that curcumin inhibits tumor growth and angiogenesis in the mouse model of human breast cancer through modulating NF- Kβ pathway (59). Also, it has a potential inhibitory effect on Wnt/β- catenin and Sonic hedgehog (shh) pathways relating to BCSCs self-renewal (60). Curcumin leads to the diminished ability of mammosphere formation and decreased the number of ALDH+ cells during consecutive passages. In contrast, curcumin poorly influences differentiated cancer cells (61). Curcumin and epigallocatechin (EPGC) could suppress the phosphorylation of STAT3, followed by decreased levels of STAT3/ NF-Kβ expression in CD44^+^ CSCs of MCF7- HER2 and MDA- MB231 cell lines (62). Curcumol is a sesquiterpenoid entity of species that belongs to the *Zingiberaceae* family including turmeric. Curcumol seems to induce cell cycle arrest at G2/M and G0/G1 phases of breast cancer cells and inhibits NF- Kβ pathway through the eliminating the protein kinase B/c-Jun N-Terminal kinases 1/2 (Akt/JNK1/2) pathway and disallowance of MMP9 (63). 

 A study investigated the role of curcumin to sensitize CSCs from MCF7 and MDA- MB231 cell lines to chemotherapy agents. Data showed that curcumin was able to reduce mammosphere formation. Also, curcumin sensitized BCSCs to mitomycin C (MMC) and the combination of mitomycin C and curcumin decreased CD44^+^/ CD24^-^ BCSCs population more than 75%. The mechanism in which curcumin sensitizes BCSCs is attributed to the decrease of ABC transporters ABCC1 and ABCG2 (64). The other mechanism of curcumin to chemosensitization of cancer cells is the up-regulation of microRNAs (miRNAs) involving in epithelial-mesenchymal transition and down-regulation of transcription factors B lymphoma Mo-MLV insertion region 1 homolog (BMI1), polycomb protein SUZ12, and enhancer of zeste homolog 2 (EZH2) (65). Curcumin could inhibit the migration and invasion activities of MDA-MB-231 cells by inhibiting the epithelial-mesenchymal transition (EMT) process. For example, curcumin was able to down-regulate the mRNA expression of β-catenin, Vimentin, and Fibronectin, while it up-regulated mRNA expression of E-cadherin. It was proved that curcumin diminishes the protein expression of Oct4, Nanog and Sox2 (66). Recently, novel strategies using curcumin have been developed. Curcumin-loaded nanoparticles in combination with 5-fluorouracil (5- FU) could inhibit cell growth and tumor progression via disturbing the E-cadherin. Also, this combination decreased lipid-peroxidation, while increased malondialdehyde (MDA) and SOD levels (67). It was shown that curcumin- doxorubicin encapsulated nanoparticles could inhibit mammosphere formation *in vitro* and tumor progression *in vivo* and decrease the number of CSCs to one fifth (68).

***Ellagic acid, ursolic acid, and luteolin***

Ellagic acid, ursolic acid, and luteolin are several polyphenolic elements of pomegranate that seem to have anti-cancer properties. Effect of standardized pomegranate extract (PE) on the CSCs isolated from the WA4 cell line shows that pomegranate extract shuts off cell cycle progression at the phase G_0_/G_1 _(69) and apoptosis induction through activating the caspase 3. Pomegranate extract components reduce the proliferation rate and viability of the WA4 cell line. It must be mentioned that all of the extracted components do not have such efficiency; for example, caffeic acid has no considerable inhibitory effect on CSCs. Another study showed that the cell cycle arrest of MCF7 cancer cells occurs due to the regulatory role of ellagic acid on the transforming growth factor-beta/Smad3 (TGFβ/ Smad3) signaling pathway (70). The combination of ellagic acid and radiation creates synergistic tumor cytotoxicity in which colony-forming potential of MCF7 cells is reduced significantly. In addition, an increased level of BAX vs decreased level of Bcl-2 pushed cancer cells into apoptosis (71). Luteolin was shown to inhibit the angiogenesis via blocking progestin-dependent vascular endothelial growth factor (VEGF) secretion by breast cancer cells. Also, it prevented medroxyprogesterone acetate (MPA)-induced acquisition state in BCSCs (72).

***Epigallocatechin***

Epigallocatechin (EPGC) is a natural compound found in several herbal extracts such as the *Spatholobus suberectus* (SS). This compound causes cell cycle arrest, progression of apoptosis and inhibition of lactate dehydrogenase A (LDH A) of MCF7 and MDA-MB 231 cell lines. Also, it inhibits LDH A expression and *in vivo* tumor growth. Inhibited expression of LDH A seems to be due to the dispersion of heat shock protein 90 (Hsp90) from hypoxia-inducible factor1α (HIF-1α) and finally acceleration of proteasome degradation of HIF-1α (73). 

In addition, Epigallocatechin gallate (EPGCG) of green tea and especially some of its analogs (analogs 4, 6) potentially activate the AMP-activated protein kinase (AMPK) in breast cancer cells. AMPK controls activities such as cell cycle progress, energy situation, protein synthesis, and cell growth and vitality. AMPK inhibits proliferation and up-regulation of an inhibitor of cyclin-dependent kinase (CDK) p21cip1. Also, it inhibits the down-regulation of the mammalian target of rapamycin (mTOR). Eventually, all of these events give rise to the suppression of the CSC population (74). 

Epigallocatechin inhibited the growth of CSCs in MDA- MB231 and MDA- MB436 cell lines and decreased the expression of estrogen receptor-α36 (75). Combination of epigallocatechin and a synthetic agonist of retinoid X receptor-γ (RXRγ), known as 6-OH-11-O-hydroxyphenanthrene (IIF), was shown to reduce expression of epidermal growth factor receptor (EGFR) and invasion- associated markers of CD44, MMP2, and 9 in breast cancer cell lines MCF7, MCF7- TAM, and MDA- MB231 (76). An *in vivo* study showed that epigallocatechin-3-gallate applies its anti- CSC activity via several mechanisms including inducing apoptosis through activation of caspase 3, inhibiting the expression of CD44 and MMP2, suppressing the angiogenesis through inhibition of VEGF and enhanced oxidative stress (77). Recently, researchers have suggested that a new derivative of EPGC, called G28, is able to inhibit the mammosphere forming potential of MDA-MB-231 cell line and its doxorubicin-resistant type. G28 inhibits the fatty acid synthase (FASN) enzyme of cancer cells, which seems to be responsible for cancer cells drug resistance (78).

***Genistein***

Soybean is a rich source of isoflavonoids among which the anti-cancer properties of genistein and formononetin (79) are well-studied. Animal studies show that soy and its genistein content reduce the quantity of mammary adipose tissue, and increase the expression of E-cadherin, mammary tumor suppressor phosphatase and tensin homolog (PTEN). Especially, it seems that genistein inhibits the differentiation of adipose tissue and in contrast, it enhances the expression of estrogen receptor β (ERβ) of the fibroblast-like cells of mammary stroma *in vivo*. In addition, genistein with similar effects decreased the amounts of CSCs and generated mammospheres *in vitro* and *in vivo*. Analyzing mechanisms of action of genistein suggest that it inhibits Akt (protein kinase B, PKB) pathway and increases PTEN expression (80). Also, MCF7 cells cultured under the genistein-treated adipocytes medium tend to form fewer mammospheres (81).

 Genistein could suppress the proliferation potential of mammospheres from the MDA- MB231 cell line through paracrine signaling pathways.  The differentiation-inducing effect of genistein seems to arise from the activation of phosphatidylinositol 3-kinase/ Protein Kinase B (PI3K/Akt) and mitogen-activated protein kinase/extracellular signal-regulated kinase (MEK/ERK) signaling pathways through paracrine factors that have been released from ER+ cancer cells (82). As a contradictory result, one study has shown that genistein induces the expression of ABCC1 and ABCG2 transporters in MCF7 and MDA- MB231 cell lines and inhibits miR-181a, an inhibitor of ABCG2 translation (83). Genistein could suppress MCF-7 CSCs proliferation *in vitro* and *in vivo* through inducing inhibitory effects on Hedgehog–Gli1 signaling pathway (84). The synergic effects of genistein and Sulforaphane (SFN) were assessed and it was found that these compounds successfully inhibited the cancer cell cycle at G2 or G1 phases. This combination could act as histone methyltransferase (HMT) and histone deacetylase (HDAC) inhibitor via down-regulating HDAC2 and HDAC3 (85). 

***Gingerol***

Gingerol is a phenolic compound existing in the oil extracted from ginger root, generating its special taste and flavor. The fresh root contains a high concentration of gingerol, while dried root bears abundant amounts of shogaol, a dehydrated form of gingerol. It has been shown that 6- shogaol could kill BCSCs effectively, inhibit mammosphere formation and increase the chemosensitivity of CSCs. Mechanism of action of 6- shogaol is down-regulation of CD44 expression, and targeting the hedgehog/ Akt/ GSK3β (Glycogen synthase kinase 3 beta) signaling pathway, leading to phosphorylation of β- catenin and decrease in expression of C- myc and cyclin D, which target stemness of BCSCs (86). It has been demonstrated that gingerol inhibits the motility, attachment, and invasion of cancer cells of the MDA-MB231cell line via an inhibitory effect on MMP2/9 activity (87).

***Icaritin***

Icaritin is a prenyl flavonoid compound isolated from *Epimedium *genus that has been used as Chinese herbal medicine for a long time. As a medication, icaritin has various properties such as protective effects on nervous system (88), stimulation of cardiac and neural differentiation (89), steroid-related osteonecrosis prevention (90), osteoblast hyperactivity and suppression of action and differentiation of osteoclasts (91) and inhibitory effect on growth of human prostate carcinoma cell line (92). 

Anti-cancer effect of icaritin in the COLO- 205 colon cancer cell line is attributed to the production of ROS, down-regulation of Bcl-2 and signaling of D1/ E cyclin, and activation of caspases 3, and 9 (93). The effect of icaritin on BCSCs is arresting the cell cycle in the G2/M phase, induction of apoptosis, and down-regulation of Bcl-2 (94). Icaritin reduces the expression of EGFR and estrogen receptor- α36 in MDA- MB231 and MDA- MB453 cells (95).

***Licochalcone E***

Another natural herbal compound that is known as a potent anti-cancer is licochalcone E (LicE), a licorice phenol derivative, that inhibits breast tumor growth and metastasis *in vitro* and *in vivo*. *In vitro* experiment showed that LicE reduced the expression of specificity protein 1 (Sp1) in MCF7 and MDA- MB231 cell lines (96). Sp1 is a protein that involves in regulating cell- cycle controlling protein as well as promoting carcinogenesis and tumor metastasis (97). 

LicE decreases the expression of cyclins and CDK and stimulates the apoptosis associated with higher expression of BAX and cloven caspases 3 and lower expression of Bcl-2. Also, LicE has other key roles including inhibition of migration and invasion of cultured cells, preventing the release of MMP9, VEGF-A, and plasminogen activator of urokinase-type and increasing the release of metalloproteinase-2 tissue inhibitor. In addition, LicE drastically limits vessel formation by endothelial cells (98).

***Noscapine (Nos)***

Noscapine is a natural alkaloid derived from *Papaver somniferum* with anti-cancer, anti-tussive, and anti-metastasis properties with low toxicity and without a sedative, addictive and analgesic properties (99). Noscapine inhibits cellular proliferation of breast, ovarian and prostate cancers (100). It was demonstrated that noscapine arrests CSCs derived from MCF-7 and MDA-MB-231 at the G2/M phase (101). It was also demonstrated that treating MDA-MB-231 cells with sub-therapeutic doses of noscapine sensitizes the cells to docetaxel through down-regulation of Bcl- 2, survivin, and pAKT, which illustrates apoptosis induction (102). Another study showed that noscapine increased BAX expression, while decreases the expression of Bcl- xl and Bcl-2. Also, it was found that noscapine initiates both extrinsic and intrinsic apoptotic pathways. Decreased level of anti-apoptotic factors may be due to decreased and increased expression of NF- Kβ and IκBα (nuclear factor of kappa light polypeptide gene enhancer in B-cells inhibitor, alpha), respectively (103). Noscapine analog seems to disrupt tubulin stability, and changes the surface configuration of tubulin fibers, preventing microtubule formation. In fact, the noscapine analog prolongs the S- phase of the cell cycle (104). Noscapine and its derivatives can interfere with the effluxion activity of P- glycoprotein (P-gp). Moreover, the combination of noscapine and vinblastine inhibited MCF7 cancer cell proliferation via tubulin dynamic disruption (105).

***Oxymatrine***

Oxymatrine is a plant alkaloid with anti-microbial (106) and anti-inflammatory (107) effects. Also, its anti-tumor effect on cancer cells is characterized by apoptosis induction and cell cycle inhibitory mechanisms (108) and an inhibitory effect on cell cycle progress following the DNA damage (109). Oxymatrine leads to a decreased rate of proliferation of MCF-7 cells, as well as a decrease in the side population (SP) cells (cancer cells with stem cell properties). The inhibitory effect of oxymatrine seems to be due to the inhibition of the Wnt/b-catenin pathway (110). Inhibition of the Wnt/b-catenin pathway leads to a suppressed Bevacizumab-induced EMT process of BCSCs (111). Chen *et al*. showed a different mechanism in which oxymatrine effectively suppressed the EMT process induced by fibronectin. Oxymatrine could hamper the α_V_β_3_ integrin/FAK/PI3K/Akt signaling pathway via inhibiting the phosphorylation of FAK (focal adhesion kinase), PI3K, and Akt (112). An *in vitro* study on MCF7 and MDA- MB231 cell lines showed that oxymatrine influences the viability and proliferation of cells in a time and dose-dependent manner. In addition, treatment with oxymatrine arrests cell cycle at S- phase, and activation of caspases 3, and 9 takes cells into apoptosis (113).

***Piperine***

Piperine is a polyphenolic compound isolated from long and black peppers. Studies on the animal model show that piperine reduces the rate of lung cancer. Recently, piperine has been used as a chemosensitizer agent in combination with rapamycin to treat breast cancer (114). Using a combination of thymoquinone and piperine in breast cancer mouse model showed significant shrinkage of tumor and occurrence of apoptosis as well as reduced levels of VEGF, suggesting the suppression of angiogenesis within the tumor (115). Also, piperine makes it possible to inhibit the Wnt/β-catenin pathway; therefore, it could be effective to target BCSCs (61). Lai *et al.* investigated the anti-cancer effect of piperine on mouse 4T1 breast cancer. They showed that piperine inhibited tumor progression in a time and dose-dependent state and induced apoptosis through activating caspase 3. Piperine made cancer cells cycle arrest at the G2/M phase with decreased expression of cyclin B1. Also, piperine prevented cancer cell migration by reducing MMP9 and MMP13 levels (116). It seems that MMP9 reduction is due to blockage of Akt, ERK1/2, and p38 MAPK signaling pathways that prevented the activation of AP-1 (activator protein 1) and NF- Kβ (117). It has been suggested that piperine could act as a pro-oxidant compound leading to ROS production, MCF7 cancer cells arrest at the G2/M phase and death (118). Moreover, piperine was able to down-regulate the expression of proteins associated with different phases of TNBC cell cycles such as cyclin D3, cyclin B, CDK4, CDK1 and p21 (119). Piperine improves anti-cancer effects of tumor necrosis factor-related apoptosis-inducing ligand (TRAIL) on MDA-MB-468 and MDA-MB-231 cell lines. Cancer cell growth suppression and apoptosis, survivin suppression and p65 phosphorylation within cancer cells reported as significant results of piperine- TRAIL combination treatment (120). 

***Pterostilbene***

Pterostilbene is a natural stilbene and dimethylated analog of resveratrol, which is found in blueberries and bears anti-cancer properties. Studies on the effect of pterostilbene on BCSCs have shown that pterostilbene induces the expression of Argonaute2 (Ago2), a kind of interfering RNA, followed by increased levels of tumor-suppressive microRNAs miR-16, miR-141, miR-143, and miR-200c. This mechanism resulted in a reduced number of BCSCs and suppressed mammosphere formation (121). This compound modulates the generation of BCSCs and tumor-associated macrophages. Tumor-associated macrophages are related to metastasis and malignancy so the co-culture of tumor-associated macrophages with cancer cells of MCF7 and MDA- MB231 cell lines is accompanied by a higher percentage of CSC content and higher expression of HIF-1α, Twist1, β- catenin and NF-Κβ transcriptional factors. Treatment with pterostilbene reduces CSC content of culture and expression of NF-Κβ, vimentin, and twist1. In contrast, the expression of E-cadherin increases. Examination of animal models shows that pterostilbene suppresses tumorigenicity and tumor metastasis (122).

 It was demonstrated that pterostilbene has anti- BCSCs effects similar to 6- shogaol. In the same way, pterostilbene could reduce BCSCs’ survival and increase the chemosensitivity of BCSCs to paclitaxel. Also, pterostilbene suppresses the mammosphere formation potential and induces membrane damage in BCSCs. Both 6- shogaol and pterostilbene share the same mechanisms to inhibit BCSCs, including decrease in the CD44 expression, and promote the phosphorylation of β- catenin as a result of suppression of hedgehog/ Akt/ GSK3β signaling pathway. Inhibition of this pathway leads to reduced expression of C- myc, and cyclin D that is associated with limited stemness potential of BCSCs (86).

***Resveratrol***

Resveratrol (Res) is a natural dietary compound found in a vast range of fruits, vegetables and red grape juice with preventive effects on cardiovascular disorders and cancer (123). The effect of resveratrol on normal cells appears as an antioxidant agent. However, in cancer cells, it has pro-oxidant and cytotoxic effects. These different effects depend on the biological characteristics of cancer cells and the unique properties of the microenvironment (124). Resveratrol seems to move intracellular copper ions, in turn contributing to Fenton reactions, leading to the production of ROS. ROS causes to oxidative break of DNA and finally cell death. Several factors, such as low PH of environment, as observed in CSCs niche, increase the number of reactions (124). 

Resveratrol could induce G1 arrest, as well as activation of caspases 8/ 9, decrease the cell viability in a dose-dependent manner in both MCF7 and MDA- MB231 cell lines, and modulate miRNAs involving in apoptosis (125). It was demonstrated that resveratrol inhibits RhoA/Lats1/YAP signaling pathway in MDA- MB231 and MDA- MB468 breast cancer cells. Inactivation of RhoA leads to activation of Lats1, which promotes the phosphorylation of YAP and finally inhibits the invasion of cancer cells. Also, a combination of resveratrol and salinomycin was known to induce mitochondria dysfunction, caspase activation followed by apoptosis, and intracellular production of ROS in four BCSC lines (126). Also, another study reported that a combination of resveratrol/ salinomycin targets the Wnt signaling pathway, which results in suppressing the viability of cancer cells, and cell cycle arrest, leading to apoptosis induction (127). Resveratrol may inhibit cell cycle at G1/ S phase in MCF7 and MDA- MB231 cancer cells through targeting Aurora protein kinase (AURKA) and the Polo-like kinase-1 (PLK1) pathways (128).  It was proved that resveratrol could alter breast tumor microenvironment through inhibiting the effects of cellular interactions on stem-like breast cancer cells. Resveratrol seemed to hamper the proliferation, migration, and invasion of breast cancer cells as well as the expression of c- myc, cyclin D1, MMP2, MMP9, CD44, SOX2 and Bmi-1 (129). 

***Isothiocyanates***

Isothiocyanates (ITCs), abundantly found in cruciferous vegetables, have an effective role to prevent human tumors. Diets containing adequate quantities of ITCs reduce the incidence risk of several cancers including lung, breast, and colon (130). Mechanisms of action for ITCs to prevent cancer are different and include activating the carcinogen detoxifying agents, and increased apoptosis versus stopped cell cycle. In addition, ITCs make it possible to suppress cell proliferation, angiogenesis, self-renewal and epithelial-mesenchymal transition of CSCs. Also, oncogene signaling pathways up-regulated in several cancers are inhibited by the ITCs. Examples of these pathways are STAT3, NF-Κβ and hormone receptors (131). One of the more known ITCs that has been studied so far is sulforaphane (SFN).

***Sulforaphane***

SFN is the main glucosinolate component of the broccoli that has been studied because of its anti-cancer effects. SFN could regulate the activity of different signaling pathways including NF-Κβ, shh, and Wnt/β-catenin. SFN has a significant role in modulating epithelial-mesenchymal transition in various types of cancers; therefore, it is regarded to target CSCs. SFN has been proposed as a chemotherapy adjuvant compound during the preclinical studies (132).

Effects of SFN on the regulation of BCSCs have been investigated *in vitro* and *in vivo*. SFN lowers the percentage of human BCSCs determined with a decreased level of ALDH+ cells and cultured mammospheres. Administration of SFN into animal models decreased the quantity of ALDH+ cells by more than 50% and inhibited the growth of cancer cells that have been cultured into the secondary hosts. The effects of SFN seemed to be applied to the down-regulation of the Wnt/β-catenin pathway that involves in self-renewal of cells (133). 

It was demonstrated that a combination of withaferin A (WA), from the Indian winter cherry, and SFN with low concentration leads to a decrease in viability and an increased apoptosis of MCF7 and MDA- MB231 cell lines. The apoptotic effect of these compounds is observed through an increased level of BAX and decreased level of Bcl- 2 (134). A similar study showed that SFN, alone or in combination with metformin (MTFN), was associated with decreased expression of Bcl-2, Wnt/β-catenin, and HER2 and increased expression of BAX in CSCs. The diminished expression of HER2 was directly correlated with cell apoptosis (135). A study showed that SFN could reduce the number of BCSCs in triple-negative breast cancer cell lines SUM149 and SUM159. SFN seems to inhibit NF- Kβ pathway. A combination of SFNwith docetaxel or paclitaxel not only increases the chemo-toxicity of chemotherapy agents but also reduces the CSC population significantly (136). SFN reduced the expression of CSCs markers including ALDH1A1, CR1, CRIPTO-3/TDGF1P3 (CR3, a homolog of CR1), Nanog, Notch4 and Wnt3. Mechanism of action in which SFN inhibits CSCs is through suppression of the Cripto/Alk4 protein signaling pathway (137). It was demonstrated that docetaxel/ SFN-loaded nanoparticles could target BCSCs and reduce their self- renewal potential. SFN nanoparticles apply their effect via down-regulating β- catenin expression (138).

***Thymoquinone***

Thymoquinone (TQ) is the main component of *Nigella sativa* seed oil with anti-inflammatory, antioxidant, and anti-cancer activity (139). The protective effects of *N. sativa* extract and TQ have been investigated against breast cancer (140). It was shown that TQ has an anti-proliferative, and pro-apoptotic effect via p38 phosphorylation and production of ROS. Also, TQ induced apoptosis in breast cancer cells via inhibiting the expression of anti-apoptotic genes like Bcl- 2, and Bcl- xL (141). It was demonstrated that TQ inhibited breast cancer cell growth through arresting cell cycle at the G1 phase, activating caspases 8, and 9 apoptotic pathways, and enhancing chemosensitivity to cisplatin and docetaxel (142). Rajput *et al*. showed that co-treatment with TQ and tamoxifen decreases the expression of X-linked inhibitor of apoptotic protein (XIAP), resulting in activation of caspase 9 apoptotic cascade. Also, breast cancer cell survival is disrupted because of inhibition of the PI3K/ Akt pathway followed by Akt phosphorylation (143). TQ augments the anti-cancer effect of gemcitabine (GCB) with an increased population of apoptotic and S phase arrested MCF-7 and T47D cell lines (144). TQ decreased the expression of Eukaryotic elongation factor-2 kinase (eEF-2K), which was associated with decreased proliferation, migration, invasion, and colony formation of triple-negative breast cancer cells both *in vitro* and *in vivo*. It seems that TQ inhibits Eef- 2k via miR603/ NF- Kβ axis; miR603 inhibits eEF-2K, while inhibition of the NF- Kβ induces the expression of miR603 (145). TQ could inhibit the metastasis of TNBC cells via down-regulating CXCR4 followed by inhibiting the NF-Κβ signaling pathway in a dose and time-dependent manner. Also, TQ treatment caused decreased migration and invasion induced by CXCL2 (146).

***Royal jelly (RJ), a novel anti-cancer agent with different origin***

Royal jelly (RJ) is a natural bee product, a white to a pale yellow jelly substance that is secreted from mandibular and hypopharyngeal glands of worker bees (147). RJ is mainly composed of water, carbohydrates, proteins, lipids, vitamins, minerals (148), acetylcholine, adenosine, adenosine monophosphate N1 oxide, polyphenols, and hormones such as progesterone, testosterone, prolactin, and estradiol (149). 10-hydroxydecanoic acid (10 HDA) and 24-methylenecholesterol are the most important bioactive compounds of RJ. 10 HDA is an unsaturated fatty acid that is only found in RJ in nature (150) and has immune-modulatory properties (151). RJ and some other compounds processed by honey bees such as propolis possess desirable therapeutic properties (152). Propolis and phenolic components of RJ have various properties including anti-inflammatory, anti-microbial, antioxidant properties and so on (152, 153). In addition to the therapeutic effects mentioned above, the anti-cancer property of RJ has been investigated in several types of cancers. It was demonstrated that 10 HDA inhibits VEGF- induced angiogenesis through both proliferation and migration of endothelial cells (154). RJ has drastic anti-proliferative potential to inhibit the proliferation of SH- SY5Y neuroblastoma cell lines (155). Recently, a study showed that RJ not only inhibited 4T1 breast cancer cells growth but also augmented the immunity of breast cancer-induced animal models indicated by the increased level of TNF- α and decreased levels of IL- 6 and IL- 10 (156). 

**Table 1:** The mechanism of action of natural herbal compounds with anti-cancer and anti-cancer stem cell effects

**Compound name**	**Kind of compound**	**Mechanism of action**	**Reference**
Apigenin	Flavonoid	ABC transporter inhibition, TNFα blockage, MMP2,9 down-regulation Apoptosis induction, cell cycle arrest STAT3 pathway inhibition, Phospho-JAK1,2 inhibition	41-47
Berberine	Alkaloid	ABC transporter inhibition, apoptosis induction, MMP2,9 down-regulation, Decreased CXCR4, CCR6, CCR9 expression	52-56
Curcumin	Polyphenol	NF-κB, Wnt/β-catenin, Shh inhibition	59-62
Curcumol	Sesquiterpenoid	Cell cycle arrest, NF-κB, Akt/JNK1/2 pathways inhibition	63 (review)
Ellagic acid	Polyphenol	Cell cycle arrest, apoptosis induction, TGFβ/Smad3 inhibition	69-71
Ursolic acid	Polyphenol	Cell cycle arrest, apoptosis induction	
Luteolin	Polyphenol	Cell cycle arrest, apoptosis induction VEGF secretion inhibition, inhibiting MPA induced acquisition state	69,72
Epigallocatechin	Polyphenol	Cell cycle arrest, apoptosis induction, HIF1α degradation, AMPK activation, decrease the expression of estrogen receptor-α36, MMP9, mTOR, FASN inhibition	73-78
Genistein	Isoflavone	Increase the expression of PTEN, PI3K/Akt, MEK/ERK, Hedgehog–Gli1 inhibition, cell cycle arrest	81,82.^84^,^85^
Gingerol	Phenol	Hedgehog/ Akt/ GSK3β pathway inhibition, MMP2,9 suppression	86,87
Icaritin	Flavonoid	Cell cycle arrest, apoptosis induction, decrease EGFR and estrogen receptor- α36 expression	94,95
Licochalcone E	Phenol	Reducing Sp1 expression, apoptosis induction, MMP9 inhibition, angiogenesis inhibition	96-98
Noscapine	Alkaloid	Cell cycle arrest and prolonged S-phase, apoptosis induction, disrupting tubulin dynamics	101,102,104,105
Oxymatrine	Alkaloid	Wnt/β-catenin, α_Ⅴ_β_3_ integrin/FAK/ PI3K/Akt inhibition, EMT suppressing Apoptosis induction	110-113
Piperine	polyphenol	Wnt/β-catenin down-regulation, Apoptosis induction, cell cycle arrest, MMP9,13 inhibition, ROS production, survivin suppression, p65 phosphorylation	61,116-120
Pterostilbene	Phenol	Inducing Argonaute2 expression Hedgehog/Akt/GSK3β suppression	86,121
Resveratrol	Phenol	Cell cycle arrest, Apoptosis induction Wnt inhibition**, **	125-128
Sulphorafane	Isothiocyanate	Wnt/β-catenin down-regulation Apoptosis induction Cripto/Alk4 suppression	133-135,137,138
Thymoquinone	Phenol	ROS production, cell cycle arrest Apoptosis induction, NF-κB inhibition	142-146

## Discussion

Breast cancer is a sophisticated disease in which treatment requires more time and cost and imposes economic and psychic pressures to the patient and the healthcare system. In addition to different therapeutic strategies and various range of drugs administered, therapy resistance and disease recurrence are regarded as a point of weakness in the treatment of breast cancer (14, 15). In recent decades, conducted studies in the field of breast cancer and introducing BCSCs theory have been accompanied by the identification of BCSCs (12), altogether have made a new era of research in front of scientists.

On the other side, in the recent years, natural herbal compounds that were thought to be applied as traditional medicine only, have been attractive for researchers who were looking for more efficient drugs to perish the CSCs. Natural herbal compounds found in fruits and vegetables that are obtained easily and in abundance could interfere with biological and metabolic pathways of CSCs, suppress these pathways, leading to inhibition of action or eradication of CSCs. Also, it must be mentioned that obtained results are on the basis of *in vivo* and *in vitro* investigations and a few numbers of these compounds have been evaluated in the form of preclinical studies (94, 132). Moreover, the anti-cancer effect of some herbal compounds such as Eupatilin on breast cancer has not been studied (157), or new targeting pathways for cancer prevention have been introduced including Malic enzyme 2 and Phosphoglycerate mutase 1 that are noteworthy (158, 159). 

## Conclusion

In closing, natural herbal compounds serve as promising therapeutic agents with multifunctional properties in prevention and inhibition of breast cancer but it is substantially suggested that the well-investigated studies be assessed in preclinical experiments with attention to ethical principles, and more experiments be designed to explore the anti-breast cancer effects of those compounds that have not been determined favorably.
